# Outcomes of viscocanalostomy and phaco-viscocanalostomy in patients with advanced glaucoma

**DOI:** 10.1007/s00417-018-4010-0

**Published:** 2018-05-22

**Authors:** M. Tsagkataki, T. M. Bampouras, A. Choudhary

**Affiliations:** 1 Royal Liverpool University Hospital, Liverpool, L7 8XP UK; 2 Active Ageing Research Group, Department of Medical and Sport Sciences, University of Cumbria, Lancaster, England

**Keywords:** Viscocanalostomy, Phaco-viscocanalostomy, Advanced glaucoma, Non-penetrating glaucoma surgery, Glaucoma

## Abstract

**Purpose:**

To determine the medium-term outcomes for patients with advanced glaucoma undergoing viscocanalostomy.

**Methods:**

All patients with advanced glaucoma (mean deviation (MD) − 12.00 dB or above) and patients with poor visual acuity secondary to advanced glaucoma which precluded formal visual field assessment undergoing viscocanalostomy (VC) and phaco-viscocanalostomy between 2010 and 2014 under the care of a single surgical team were included. Intraocular pressure (IOP), visual acuity (VA) and visual field outcomes were assessed from data prospectively collected into a surgical outcome database. Success was defined at two IOP cut-off points: IOP ≤ 21 and ≤ 16 mmHg with (qualified) or without (complete) medications.

**Results:**

One hundred thirty-five patients were included. Mean IOP changed from 23.6 ± 6.4 mmHg pre-operatively to 15.3, 15.8 and 14.8 mmHg at 1, 2 and 3 years, a change of 35, 33.5 and 39% respectively. Qualified success for an IOP ≤ 21 mmHg was achieved in 95.66, 90.6 and 80% and complete success in 52.5, 48.6 and 30.6% at year 1, 2 and 3. Qualified success for an IOP ≤ 16 mmHg was achieved in 66.6, 66.05 and 60% and complete success in 44.8, 37.6 and 30.6% at year 1, 2 and 3. The cumulative probability for achieving an IOP ≤ 21 mmHg with or without drops was 86.1, 81.4 and 81.4% at 12, 24 and 36 months. Eleven patients (8.1%) failed to achieve adequate IOP control and needed further surgical intervention. Eleven (8.1%) patients needed an intervention (Yag goniopuncture) following VC. Four patients (2.9%) had some post-operative complications, which resolved within 2 weeks following surgery. Nine patients (6.7%) lost more than 2 Snellen lines. There was no significant change in the MD across time points.

**Conclusion:**

Viscocanalostomy and viscocanalostomy combined with phacoemulsification is a safe and effective method of controlling IOP in the medium term in patients with advanced glaucoma.

## Introduction

Patients most at risk of blindness during their lifetime, due to glaucoma, are those who present with advanced disease. Almost 60% of patients progressing to statutory blindness have one eye with an MD worse than − 14 dB at baseline [1]. Ten to thirty-nine percent of glaucoma patients present with advanced disease in at least one eye in the UK [2–4]. The preferred option for most glaucoma and non-glaucoma specialists in the UK is to start with primary medical therapy, citing surgical risk as the primary reason behind it (23 and 22% respectively) [5].

National Institute for Health and Clinical Excellence (NICE) guidelines recommend primary surgery for patients presenting with advanced disease [6]. However, there is limited evidence supporting this recommendation and the type of surgery to be offered.

Stead and King [7] have reported medium-term results for trabeculectomy combined with mitomycin C (MMC) in patients with advanced glaucoma (MD ≤ 20 dB). Although trabeculectomy was successful at controlling intraocular pressure (IOP) in this group, a quarter of patients experienced a significant reduction in acuity, with the pre-operative MD cited as the only determinant for it.

The treatment for advanced glaucoma study (TAGS) will report the outcomes of primary trabeculectomy augmented with MMC compared with medical management for advanced glaucoma [8]. Non-penetrating glaucoma surgery (NPGS) has been shown to provide comparable long-term qualified success rates to trabeculectomy, with reduced post-operative complications [9–11] but there is limited information on its success in patients with advanced glaucoma.

In this study, we assessed the outcomes of viscocanalostomy (VC) and phaco-viscocanalostomy (phaco-VC) in patients with advanced glaucoma to document success in terms of IOP and VA and the post-operative interventions undertaken to achieve these.

## Methods

Advanced glaucoma was classified as MD between − 12.00 and − 20.00 dB and severe glaucoma MD − 20.01 dB or worse [12].

All patients with advanced glaucoma and patients with poor VA secondary to advanced glaucoma which precluded formal visual field (VF) assessment undergoing VC and phaco-VC between 2010 and 2014 under the care of a single surgical team were included.

The technique involved superior corneal traction with 7-0 vicryl. A fornix-based conjunctival flap was raised superiorly and haemostasis was achieved using wet field cautery. A two-third scleral thickness limbus-based flap (5 × 5 mm) was raised and advanced 1 mm into clear cornea. A 4 × 4-mm deep scleral flap was dissected to the level of Schlemm’s canal (SC), de-roofing it, and extended into corneal stroma to the level of Descemet’s membrane to create the trabeculo-Descemet’s membrane (TDM). Viscoat™ (sodium hyaluronate and sodium chondroitin sulphate) was injected into the two surgically created ostia of SC, aiming at dilating both the ostia and the canal, and was also placed in the scleral bed. The deep flap was excised close to the TDW. The superficial scleral flap and conjunctiva were closed tightly with 10-0 vicryl sutures. The formation of a bleb was not intended. No antimetabolite was used.

There were no specific exclusion criteria. Data on all patients were included until the last recorded appointment, which was considered the end of their follow-up. Post-operative time points analysed were day 1, week 1, month 3, month 6 and then every 6 months or closest to that point. Minimum follow-up was 1 year. An intervention was defined as any procedure or process undertaken after VC aimed at enhancing the success of the surgical outcome. This was Nd:YAG laser goniopuncture (Yag GP).

Primary outcomes were changes in IOP and visual field (MD). These were assessed from data prospectively collected into a surgical outcome database. Secondary outcomes were change in VA, post-operative complications and interventions and number of glaucoma drops (Drops) used.

Subgroup analysis was performed to look at confounding factors such as age, race, combined cataract surgery and previous glaucoma surgery or laser. Differences in outcomes between primary open-angle glaucoma (POAG) and secondary glaucoma were also examined.

SPSS v22 (SPSS, Chicago, IL) was used to perform statistical analysis. Normality of data (IOP, Drops and visual field MD) was examined using the Kolmogorov-Smirnov test. A linear mixed model was used to examine for differences in IOP and MD between pre-operative (pre-op) and year 1 (Y1), year 2 (Y2), year 3 (Y3) post-operatively and to compare the two MD groups at these time points. Drops were examined with Friedman’s test and if a difference was found, pairwise comparisons were conducted using the Wilcoxon test. Associations between presenting IOP, cataract surgery post-VC and combined phaco-VC were examined with point biserial correlation.

### Success definition

Success was defined at two IOP cut-off points (IOP ≤ 21 mmHg and IOP ≤ 16 mmHg). Complete surgical success was IOP ≤ 21 or ≤ 16 mmHg with no additional medications and qualified surgical success with additional glaucoma medications. Failure was defined as IOP **>** 21 mmHg on two consecutive visits, IOP ≤ 5 mmHg on two consecutive visits after 3 months, reoperation for glaucoma or loss of light perception. VA was measured on a Snellen chart, and a reduction of ≥ 2 lines was considered clinically significant.

## Results

One hundred thirty-five eyes of 133 patients were included in the study. Patient demographics for all variables over time can be seen in Table 1. Sixty-three (46.7%) patients had phaco-VC. The majority of eyes (132) were on topical glaucoma drops pre-operatively. IOP at diagnosis was not known in 55 patients, as these were referred from other units, and this information was not provided. Twenty-five patients had had some previous intervention for glaucoma including trabeculectomy, cyclodiode laser, argon laser trabeculoplasty (ALT) and selective laser trabeculoplasty (SLT) (Table 1).

**Table 1:** Patient demographics

	*N* (%)	Mean	SD	Median	Range
Age (years)	135 (100)	69.8	13.9	73	25 to 92
Duration of glaucoma (years)	101 (74.8)	10.8	7.1	10	1 to 30
Race					
Caucasian	125 (92.6)				
African/Afro-Caribbean	4 (3)				
Asian	6 (4.4)				
Presenting intraocular pressure (mm Hg)	78 (57.8)	32.7	12.0	29	17 to 64
Pre-operative intraocular pressure (mm Hg)	135 (100)	23.6	6.4	22	14 to 44
Pre-operative visual acuity	111 (82.2)			6/6–6/9	6/6–6/9 to hand movement
Pre-operative drops (number)	135 (100)	3.1	1.1	3	0 to 5
Pre-operative MD (dB)	115 (85.2)	− 19.6	5.5	− 19.4	− 12 to − 32.7
Previous procedures					
None	110 (81.4)				
Trabeculectomy	11 (8.1)				
Retinal detachment surgery	7 (5.2)				
SLT, ALT	4 (3.0)				
Cyclodiode	3 (2.2)				
Glaucoma type					
Primary open-angle glaucoma	92 (68.1)				
Chronic angle closure glaucoma	11 (8.1)				
Pseudoexfoliation	10 (7.4)				
Uveitic	8 (5.9)				
Pigment dispersion	6 (4.4)				
Normal tension glaucoma	5 (3.7)				
Fuchs’ heterochromic cyclitis	3 (2.2)				

Patient numbers (and as percentage of whole sample), mean ± SD or median and range (minimum to maximum values), as appropriate, are presented for each variable

### IOP outcome

**Fig. 1:**
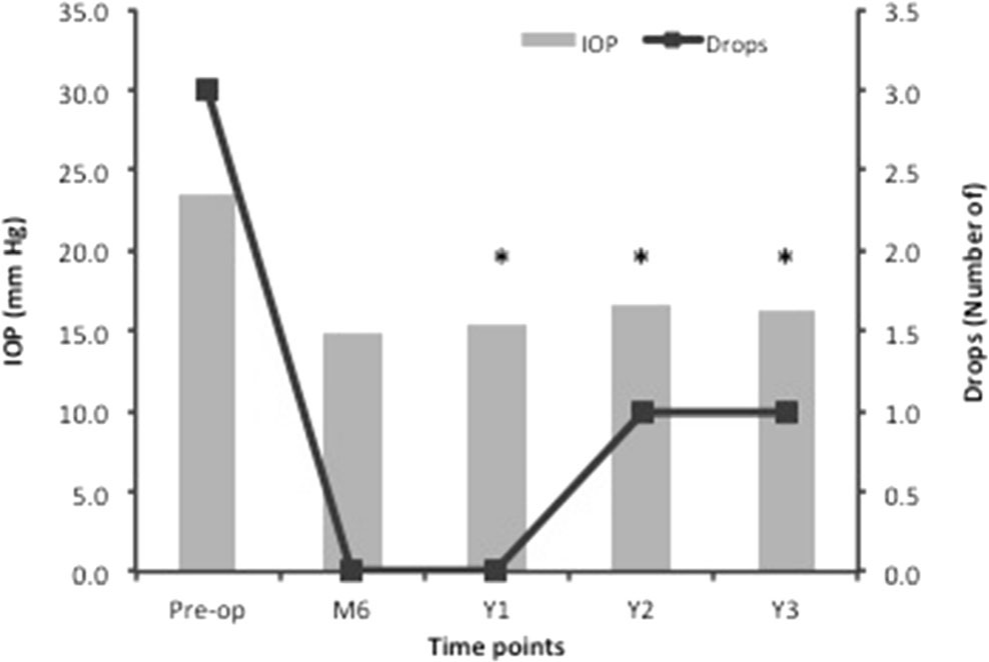
Mean intraocular pressure (IOP) and median number of drops (Drops) plotted against the time points. Error bars have been excluded for clarity. Asterisk denotes significant difference with pre-operative values. Pre-op, pre-operative; M6, month 6 post-operative (not considered in statistical analysis); Y1, year 1 post-operation; Y2, year 2 post-operation; Y3, year 3 post-operation

IOP was significantly lower at all examined time points (Y1 by 35.0%; Y2 by 33.5%; Y3 by 39.2%, *p* < 0.001 at all time points) compared to the pre-op value. Glaucoma drops were significantly different across time points (*p* = 0.001) with a significantly lower median at all examined time points compared to pre-op. Visual field MD was not significantly different across time points (*p* = 0.289). When comparing IOP, Drops and MD scores between the two MD groups, no significant interaction was seen between groups and time points for IOP (*p* = 0.999), Drops (*p* = 0.384) or MD (*p* = 0.061). Descriptive statistics of all of the above can be seen in Table 2, while Fig. 1 displays IOP and Drops data plotted over the time points.

**Table 2:** Descriptive statistics of IOP, Drops and MD at all time points including number of patients per year

	Pre-op (135)	Y1 (135)	Y2 (109)	Y3 (75)
IOP (mm Hg)	23.6 ± 6.4	15.3 ± 3.2*	15.8 ± 4.1*	14.8 ± 3.4*
For MD between − 12.01 and − 20.00 dB	22.1 ± 5.2	15.3 ± 3.1	15.9 ± 3.3	15.0 ± 2.7
For MD − 20.01 dB and worse	23.9 ± 6.5	15.5 ± 3.6	15.2 ± 3.1	14.8 ± 3.8
Drops (number of)	3, 0–5	0, 0–3*	1, 0–4*	1, 0–4*
Mean deviation (dB)	− 19.6 ± 5.5	− 18.8 ± 5.6	− 18.0 ± 10.5	− 19.8 ± 4.4

IOP and MD data is presented as mean ± SD, while Drops data as median and range. Asterisk denotes significant difference with pre-op

*IOP* intraocular pressure, *Drops* number of medications, *MD* mean deviation, *Y1* year 1 post-operation, *Y2* year 2 post-operation, *Y3* year 3 post-operation

Qualified success for an IOP ≤ 21 mmHg was achieved in 95.66, 90.6 and 80% and complete success in 52.5, 48.6 and 30.6% at year 1, 2 and 3. Qualified success for an IOP ≤ 16 mmHg was achieved in 66.6, 66.05 and 60% and complete success in 44.8, 37.6 and 30.6% at year 1, 2 and 3 (Table 3).

**Table 3:** Complete and qualified success for IOP ≤ 16 mmHg and IOP ≤ 21 mmHg (number and percentage)

	Y1	Y2	Y3
IOP ≤ 16 mmHg with no medication	60/135 (44.8%)	41/109 (37.6%)	23/75 (30.6%)
IOP ≤ 16 mmHg with additional medication	90/135(66.6%)	72/109 (66.05%)	45/75 (60.0%)
IOP ≤ 21 mmHg with no medication	71/135 (52.5%)	53/109 (48.6%)	23/75 (30.6%)
IOP ≤ 21 mmHg with additional medication	129/135(95.66%)	96/109 (90.6%)	60/75 (80%)

*IOP* intraocular pressure, *Y1* year 1 post-operation, *Y2* year 2 post-operation, *Y3* year 3 post-operation

Eleven patients (8%) failed (4 in Y1, 4 in Y2 and 3 in Y3) to reach any of the above success criteria and needed further surgical intervention (Table 4). Of these, four had uveitic glaucoma, two were pseudoexfoliative glaucoma (PXFG) and five were POAG.

**Table 4:** Details of failures

No	Type of glaucoma	Previous surgery/laser	Pre-op IOP (mmHg)	Pre-op MD (dB)	Pre-op BCVA	Pre-op drops (No)	BCVA at final follow-up	MD (dB) at final follow-up	Time of failure post-op (months)
1	FHC	Nil	20	U/C	6/60	3	6/60	U/C	24
2	FHC	Nil	32	− 18.59	6/12	3	6/18	− 21.54	36
3	POAG	Nil	28	− 25.7	6/6	1	6/9	− 29.23	36
4	PXFG	Nil	39	− 27.48	6/36	5	6/36	U/C	24
5	POAG	Previous VC	30	− 12.87	6/6	4	6/9	− 14.85	24
6	FHC	Nil	28	− 23.92	6/12	4	6/18	− 25.48	24
7	POAG	Nil	24	− 21.73	6/6	4	6/9	− 22.68	12
8	POAG	Nil	24	U/C	6/60	4	6/60	U/C	24
9	Uveitic glaucoma	Cyclodiode	28	− 14.73	6/24	4	6/36	− 16.73	24
10	POAG	Nil	25	− 16.73	6/6	4	6/6	− 17.75	12
11	PXFG	Trabeculectomy	16	− 21.07	6/12	4	6/12	− 22.78	24

*U/C* unable to perform reliable fields, *POAG* primary open-angle glaucoma, *FHC* Fuchs’ heterochromic cyclitis, *PXFG* pseudoexfoliation glaucoma

**Fig. 2:**
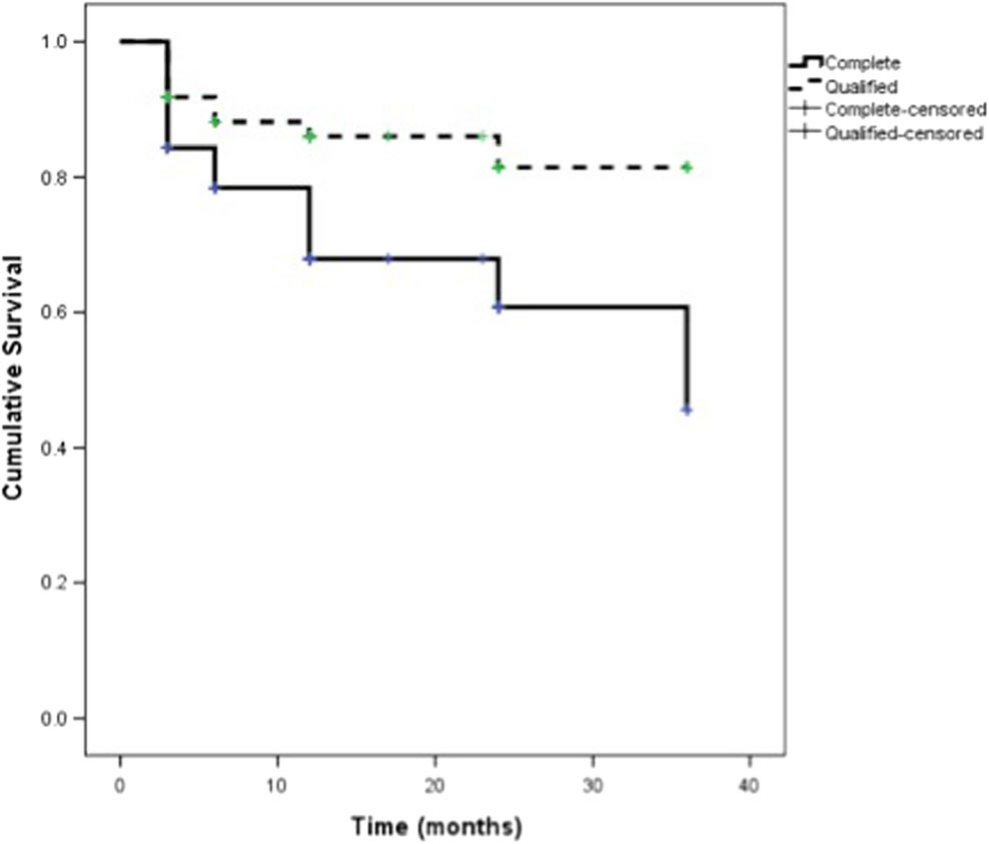
Kaplan-Meier survival plot for complete (defined as IOP ≤ 21 mmHg with no medication) and qualified (defined as IOP ≤ 21 mmHg with additional glaucoma medications) success

Kaplan-Meier survival curves were significantly different between complete and qualified success with IOP ≤ 21 (*p* = 0.001, Fig. 2) but not between different glaucoma types (complete (*p* = 0.912) and qualified (*p* = 0.541)) or between MD groups (complete (*p* = 0.512) and qualified (*p* = 0.079)).

### Visual acuity outcome

VA was stable for the vast majority of patients (126 patients, 93.3%). Nine patients lost > 2 Snellen lines. This was from glaucoma and high myopia in seven and proliferative diabetic retinopathy in two patients. Majority of these patients (eight out of nine) had MD worse than − 20 dB. In two patients with MD < − 20 dB, VA dropped significantly from 6/36 at pre-op to hand movement and perception of light in the early post-operative period (presumed wipe out).

### Visual field changes

One hundred fifteen patients were able to perform a reliable VF (24-2 Humphrey’s visual field) prior to surgery. The number of patients that were able to perform a reliable VF gradually decreased over the follow-up period. Fifty-two patients were able to complete a reliable VF by final follow-up. In these patients, MD was not significantly different across time points (*p* = 0.105) compared to pre-op as a group and for individual patients.

### Confounding factors

Presenting IOP, age, glaucoma type and glaucoma duration and having previous surgery did not comprise a sufficiently good model that could predict the intervention (Yag GP, *p* = 0.128) or intra- and post-operative complications following surgery (*p* = 0.175). There was no significant difference noted in the outcome between VC and phaco-VC (*p* = 0.313). All descriptive statistics of the above variables can be found in Table 1.

### Cataract surgery

Three (5%) out of the 59 patients, who underwent VC and were phakic at the time of surgery, underwent cataract surgery by their final follow-up. Twenty-three (17%) were pseudophakic pre-viscocanalostomy.

### Complications

Four patients (2.9%) had some post-operative complication following VC, which resolved within 2 weeks and did not cause any visual loss (Table 5).

**Table 5:** Intra- and post-operative complications

Complication type	No. of patients
Intra-op TDW* perforation	17/135
Wound conjunctival leak treated with bandage contact lens	2/135
Scleral flap leak repaired with tutoplast	1/135
Wound leak repaired with suturing	1/135

*Trabeculo-Descemet’s window

### Post-operative intervention

Eleven (8.1%) patients needed Yag GP following their operation. The time frame for this varied between 2 and 18 months.

## Discussion

We report our results with un-augmented VC in a cohort of patients with advanced glaucoma. VC was able to achieve an IOP ≤ 21 mmHg in 80 (3 years) to 95% (1 year) patients with a 35–39% drop in IOP from baseline with a good safety profile. To our knowledge, our study provides the largest number of eyes with the longest follow-up yet reported for VC in patients with advanced glaucoma.

There is limited evidence for the outcomes of glaucoma surgery for advanced glaucoma and no recent studies reporting the outcomes of NPGS for this cohort of patients. Ates et al. [13], in 1999, reported their experience of deep sclerectomy with collagen implant in 54 eyes with advanced glaucoma with 96.2% patients maintaining an IOP < 18 mmHg over 2 years. In our cohort, IOP reduction was maintained significantly below pre-operation levels up to 3 years after surgery. We have previously reported qualified (87.5–90.2%) and complete success (78–90%) rates in a cohort of patients with POAG [10]. Shaarawy et al. [11] have previously reported a 90% qualified and 60% complete success rate at 5 years with VC. It is likely that Schlemm’s canal sclerosis and collapse with advanced disease is the most likely explanation for the lower complete success rates for VC in the present study. We have previously augmented VC with MMC in high-risk eyes [9] but did not find a difference in outcome when compared to un-augmented VC [9, 10], which suggests a possible bleb-independent mechanism for the success of VC.

Trabeculectomy is still considered the gold standard and achieves better control of IOP than VC [14]. The benefits of NPGS however are potential gains for the patient in terms of their quality of life and reduced likelihood for post-operative interventions and sight-threatening complications [14]. Kirwan et al. [15] in a recent multicenter analysis of current trabeculectomy practice reported the requirement for frequent post-operative interventions in the majority of patients concluding that completion of trabeculectomy is just the beginning of a process that takes several months to complete.

There are no like-for-like trials and limited published data to compare our results to those for trabeculectomy or NPGS in a similar patient cohort. Stead and King’s [7] results for trabeculectomy augmented with MMC in advanced glaucoma fare better in terms of IOP control compared to our group. However, with regard to post-operative interventions, 79.8% patients had some form of bleb manipulation [7] compared to only 8.1% in our study that had Yag GP.

Reduced VA is a well-recognised complication of glaucoma surgery and might be due to glaucoma progression, comorbidity or the procedure itself. Kirwan et al. [15] reported the outcomes of 428 trabeculectomies. Fifteen percent had lost > 1 Snellen line at 1 year and 6% had lost > 2 Snellen lines by 2 years post-trabeculectomy (13% with advanced visual field loss). Twenty-seven percent of patients in Stead and King’s study experienced a loss of two or more lines of Snellen acuity [7]. Nine patients (6.7%) in our study experienced a loss of > 2 Snellen lines. Eight of these nine patients had a MD worse than 20 dB. The drop in vision was attributed to glaucomatous progression in seven eyes (5.2%), two (1.5%) of which were presumed to be a wipe out. The risk of loss of central vision in patients with advanced VF loss ranges from rare to as high as 14% [16, 17]. This may be attributable to readily identifiable complications including cataract, cystoid macular edema, suprachoroidal and vitreous haemorrhage, endophthalmitis and uveitis or be unexplained (wipe out). The exact mechanism of the “wipe out” phenomenon remains elusive, but has been linked to sudden intra-operative hypotony resulting in optic nerve haemorrhage and decreased perfusion pressure to an already compromised nerve [18]. The lower rates of drop in vision in the present study reflect the benefit of avoiding sudden decompression with VC in eyes with end-stage glaucoma.

Cataract formation is a reported complication after trabeculectomy and can be in the order of 78% [19]. More recent data suggest this to be in the order of 30% [15]. King et al. [7] reported a 63% incidence of cataract requiring surgery in 27% cases. In comparison, only 5% patients in our group required cataract surgery in the study period. Cataract surgery can decrease the success of a trabeculectomy with an increased likelihood of post-operative interventions and requirement for glaucoma medication in 30–39% cases [20, 21]. These risks are avoided with VC due to its bleb-independent mechanism of action.

NICE recommends primary surgery should be offered to patients presenting with advanced loss [6]. Stead and King recommend this to be trabeculectomy augmented with MMC [7]. The use of antimetabolites is a recognised risk factor for bleb-related infection and endophthalmitis [22] and reflects the opinion of UK Consultants’ for not advocating primary surgery for this group [2]. Avoidance of antimetabolite use and bleb-related complications with VC is an important consideration in this group of patients. An advantage of VC is retention of the TDW, which appears to serve as a barrier to infection [22] and allows titrated aqueous flow, thus avoiding hypotony and its complications.

NPGS techniques have greater safety with a lower risk of complications when compared to trabeculectomy [22–25]. A Cochrane review reported relatively fewer complications with NPGS (17%) compared to trabeculectomy (65%) [14]. The UK national trabeculectomy surgery survey [26] reported early complications in 46.6% and late complications in 42.3% cases. Only four patients (2.9%) had some post-operative complication in our study, which resolved within 2 weeks with no long-term complications.

Visual field MD was not significantly different across time points (*p* = 0.105) compared to pre-op in our study. However, the number of patients completing a VF declined year on year, with 45.2% of patients finally completing a reliable VF. This may be expected with time [1] and is comparable to previous reports where only 39% of patients with advanced glaucoma were able to complete a reliable VF at 1 year, 35% at 3 years and 17% at 5 years post-trabeculectomy [7, 27].

Of the 11 eyes that failed, 4 had uveitic glaucoma (3 Fuchs’ heterochromic cyclitis (FHC)) and 2 PXFG) both known to have an aggressive course [28]. We have previously reported good outcomes for NPGS in uveitic glaucoma [10]. In this study, eight patients had uveitic glaucoma of which three had FHC, all of which failed. This could be related to the increased likelihood of subclinical neovascularisation and Schlemm’s canal sclerosis in FHC [29].

Limitations of this study include the loss of numbers, particularly those able to complete a reliable VF test during the follow-up period. However, to our knowledge, this is the first study to report the effectiveness of un-augmented VC for advanced glaucoma. IOP remained controlled over a period of up to 3 years, albeit with the requirement of increasing medications with time and VA remained stable in the majority of patients. VC had similar qualified success rate to MMC trabeculectomy with a good safety profile, avoidance of MMC and its attendant complications and minimal post-operative interventions. The benefits can also be extrapolated to the wider context of economic and quality of life benefits to be achieved with NPGS [30]. NICE recommends primary surgery in this group of patients [6]. TAGS will address the outcomes of primary trabeculectomy with MMC for advanced glaucoma [8]. Our study supports the extension of the trial to include the use of primary VC for advanced glaucoma.
